# Unlocking the connection: hemoglobin glycation index as a key driver of bone loss in diabetes-related osteoporosis

**DOI:** 10.3389/fendo.2025.1574064

**Published:** 2025-06-24

**Authors:** Zhangxin Wen, Xinpeng Liu, Wanlin Jin, Zhi-Feng Sheng, Hong Liu

**Affiliations:** ^1^ Department of Metabolic Endocrinology, Zhuzhou Hospital Affiliated to Xiangya School of Medicine, Central South University, Zhuzhou, China; ^2^ National Clinical Research Center for Metabolic Diseases; Health Management Center and Department of Metabolic Endocrinology, Xiangya Second Hospital, Central South University, Hunan Key Laboratory of Metabolic Bone Diseases, Changsha, China

**Keywords:** hemoglobin glycation index (HGI), vertebral bone mineral density, osteoporosis, bone resorption, bone formation

## Abstract

**Background:**

Osteoporosis, marked by decreased bone density and heightened fracture risk, is prevalent in aging individuals with type 2 diabetes mellitus (T2DM). The hemoglobin glycation index (HGI), a novel biomarker for glycation status, reflects advanced glycation end products (AGEs) accumulation. However, its role in bone metabolism and osteoporosis development remains poorly understood.

**Methods:**

We enrolled 412 hospitalized T2DM patients to investigate the relationship between HGI and vertebral bone mineral density (BMD). BMD was measured by dual-energy X-ray absorptiometry (DXA), and bone turnover markers (PINP, β-CTX, OC) were evaluated. Correlation analyses were conducted to explore the associations between HGI, BMD, and bone cell activity markers. Mediation analysis was performed to determine whether osteoclast activity mediated the relationship between HGI and vertebral BMD.

**Results:**

Patients with vertebral fractures exhibited significantly higher HGI levels compared to those without fractures (0.8 ± 2.1 vs. 0.3 ± 2.1, respectively). A negative correlation was observed between HGI and vertebral BMD (r = -0.140, p = 0.005), while HGI showed a positive correlation with CTX (r = 0.15, p = 0.03). No significant association was found between HGI and P1NP (r = 0.022, p = 0.755). Mediation analysis revealed that osteoclast activity accounted for 28.88% of the relationship between HGI and vertebral BMD. Further subgroup analysis by age (<65 and ≥65 years) indicated that the association between HGI and vertebral BMD was stronger in patients aged ≥65 years, suggesting age-related differences in the HGI-osteoporosis relationship.

**Conclusion:**

This study demonstrates that HGI contributes to bone loss and reduced vertebral BMD by enhancing osteoclast activity. While the impact of HGI on osteoblast function remains unclear, its significant influence on bone resorption highlights its potential role in the pathogenesis of osteoporosis in T2DM patients. These findings offer novel insights into the relationship between diabetes and osteoporosis and suggest that managing HGI levels may provide a therapeutic target for preventing osteoporosis and fractures in T2DM patients.

## 1 Introduction

Osteoporosis is a chronic condition characterized by reduced bone mass and deterioration of bone microarchitecture, resulting in increased bone fragility and susceptibility to fractures (1). This condition is a major global health concern, particularly in aging populations, and is associated with significant morbidity, reduced quality of life, and economic burden (2). This condition is notably more prevalent among individuals with type 2 diabetes mellitus (T2DM) (3), highlighting the intricate relationship between metabolic disorders and skeletal health. Bone mineral density (BMD), commonly measured using dual-energy X-ray absorptiometry (DXA), is the primary diagnostic tool for assessing osteoporosis and fracture risk. However, recent studies suggest that bone quality, bone turnover markers (BTMs), and hormonal factors may also play essential roles in understanding the pathophysiology of osteoporosis and developing effective interventions (4–6). T2DM-related osteoporosis not only increases the risk of fractures and associated complications but also underscores a critical need to unravel the mechanisms underlying this relationship (7). Notably, individuals with diabetes often have normal or even higher bone mineral density (8), making it challenging to predict fracture risk solely through bone density assessments. Despite extensive research, the precise pathways connecting diabetes and bone health remain poorly understood, hindering the development of effective strategies to reduce osteoporosis risk in this vulnerable population.

Hemoglobin glycation index (HGI), a measure of individual variation in glycation relative to average blood glucose levels (9), has been extensively studied in the context of diabetes and its complications (10–12). Studies have proven that HGI in nondiabetic patients is positively associated with risk factors of metabolic diseases, such as hepatic steatosis (13), hypertension (14), and coronary artery calcification (15). HGI may also influence bone health, because HGI is positively associated with the accumulation of advanced glycation end-products (AGEs) (16)and chronic hyperglycemia and advanced glycation end-products (AGEs) can impair bone matrix properties, increase oxidative stress, and alter bone remodeling processes, leading to decreased bone strength (17, 18). This effect contribute to reduced BMD and increased fracture risk. Given this evidence, HGI may serve as a key mediator linking glycation to skeletal health. While previous studies have explored the relationship between diabetes and osteoporosis, the mechanisms through which HGI influences bone health and its differential impact across age groups remain poorly defined.

Bone turnover markers (BTMs) are biochemical indicators of bone remodeling activity and are categorized into markers of bone formation (19), such as procollagen type I N-terminal propeptide (PINP), and markers of bone resorption, such as C-terminal telopeptide of type I collagen (CTX). These markers provide insights into the dynamic process of bone metabolism (20), which cannot be reflected by fixed measures like BMD. Understanding the interaction between HGI, BTMs, and BMD may offer novel perspectives on the mechanisms underlying bone fragility and help identify at-risk populations for targeted interventions.

This study aims to bridge the gap in literature by exploring how HGI correlates with BMD and fracture at various skeletal sites, as well as its relationship with BTMs in a population-based cohort. By elucidating these relationships, we seek to enhance the understanding of metabolic influences on bone integrity in diabetic populations and seeks to provide insights into novel pathways underlying osteoporosis development and identify potential targets for preventive and therapeutic interventions.

## 2 Patients and methods

### 2.1 Study population

The patients were evaluated in the Zhuzhou central Hospital. We collected data from 412 hospitalized diabetes patients between 2019 and 2023, consisting of males over 50 years old and postmenopausal females. Patients were excluded if they had type 1 diabetes mellitus, any acute inflammation, active infection, cancer, chronic liver diseases and severe renal impairment and an eGFR less than 30. This research was approved by the ethics committee, and written informed consent was obtained from all enrolled patients.

### 2.2 Clinical examination and biochemical analysis

All subjects underwent dual energy X-ray bone densitometry to assess the T-score of BMD (DEXA, Hologic, France). The participants’ systolic blood pressures (SBP) and diastolic blood pressures (DBP) were measured after a 5-min rest using a sphygmomanometer. Venous blood samples were drawn after an overnight fast. All biochemical analyses were performed in our hospital, including a routine blood test, fasting blood glucose (FPG), fasting C-peptide (FC-p), osteocalcin (OC), serum creatine(Cr), 25-OH vitamin D3 (VitD3), total cholesterol (TC), triglyceride (TG), low density lipoprotein cholesterol (LDL-C), C-terminal telopeptide of type I collagen (β-CTX), procollagen type 1 N propeptide (P1NP), high-density lipoprotein cholesterol (HDL C), and glycosylated hemoglobin A1c (HbA1c).

### 2.3 Statistical analysis

Non-parametric analyses were used to compare non-normally distributed numerical variables, and the results were expressed as the median and interquartile range. Logistic regression was used to analyze Osteoporosis in postmenopausal females with T2DM with risk factors. The relationship between these two was investigated further several sensitivity analyses. The present study employed curve fitting techniques to investigate the potential association between the HGI index and BMD. The existence of a non-linear connection was demonstrated, Subgroup analyses were then stratified using logistic regression models according to factors such as gender, age, Mediator analysis was used to determine the possible mediated influence of Bone formation markers on the relationship between the HGI and Spine BMD. The impact of the HGI on Spine BMD in the absence of mediators is known as the direct effect (DE). CTX indirect effects (IE), which are mediated by mediators, are the results of the Spine BMD. By dividing IE by TE (total effect), the proportion of mediators was calculated. We performed bootstrapping with 1,000 resamples to validate the mediation analysis results. Gradient Boosting Machine (GBM) model were used to analyze the importance of each feature affecting BMD. Vertebral fractures were radiographically confirmed using Genant’s semi-quantitative method. Analyses were performed using SPSS 27.0.and p<0.05 was considered statistically significant.

## 3 Results

### 3.1 Comparison of characteristics between the osteoporosis and non-osteoporosis groups

In the comparison between the osteoporosis and non-osteoporosis groups, significant differences were observed in bone mineral density (BMD) across multiple sites, including L1BMD(P < 0.001), L2BMD, L3BMD, Spine BMD, femoral neck BMD, Hip BMD and L4BMD (P < 0.001). The osteoporosis group exhibited significantly lower BMD compared to the non-osteoporosis group. Furthermore, the major osteoporotic fracture (MOF) and hip fracture (HF) risks were substantially higher in the osteoporosis group than in the non-osteoporosis group (P < 0.001). Body mass index (BMI) also differed significantly between the two groups (P = 0.008), with lower BMI observed in the osteoporosis group (**Table 1**).

**Table 1:** Demographic characteristics of the osteoporosis group and the non-osteoporosis group.

Osteoporosis	1	2	Standardize diff.	P-value
N	111	301		
Age	66.5 ± 9.2	62.9 ± 8.4	0.4 (0.2, 0.6)	<0.001
eGFR	109.1 ± 57.4	108.2 ± 38.0	0.0 (-0.2, 0.2)	0.862
Creatine	72.6 ± 28.5	72.0 ± 27.0	0.0 (-0.2, 0.2)	0.833
WC	86.5 ± 10.1	88.5 ± 9.0	0.2 (-0.0, 0.4)	0.057
HbA1C	9.3 ± 2.1	9.1 ± 2.4	0.1 (-0.1, 0.3)	0.409
FPG	9.0 ± 3.1	8.7 ± 3.1	0.1 (-0.1, 0.3)	0.418
TG	1.7 ± 1.3	2.1 ± 1.8	0.2 (-0.0, 0.4)	0.081
SHR	0.8 ± 0.2	0.8 ± 0.2	0.0 (-0.2, 0.3)	0.736
TyGindex	9.2 ± 0.8	9.3 ± 0.8	0.2 (-0.1, 0.4)	0.142
BMI	23.9 ± 3.4	24.9 ± 3.4	0.3 (0.1, 0.5)	0.008
MOF	6.5 ± 3.3	3.1 ± 1.4	1.4 (1.1, 1.6)	<0.001
HF	3.8 ± 4.1	0.9 ± 0.9	1.0 (0.7, 1.2)	<0.001
PINP	48.2 ± 21.9	47.4 ± 21.7	0.0 (-0.3, 0.4)	0.825
Vitamin D	56.6 ± 41.2	53.2 ± 17.3	0.1 (-0.1, 0.3)	0.250
CTX	437.6 ± 242.9	431.9 ± 254.3	0.0 (-0.3, 0.3)	0.890
OC	16.2 ± 15.7	14.3 ± 6.4	0.2 (-0.2, 0.5)	0.219
Ca	2.2 ± 0.2	2.3 ± 0.1	0.1 (-0.2, 0.4)	0.481
AIP	0.1 ± 0.3	0.2 ± 0.3	0.2 (0.0, 0.5)	0.046
L4BMD	0.8 ± 0.2	1.1 ± 0.3	1.1 (0.7, 1.4)	<0.001
L3BMD	0.8 ± 0.2	1.0 ± 0.2	1.4 (1.1, 1.8)	<0.001
L2BMD	0.7 ± 0.1	1.0 ± 0.2	1.6 (1.2, 1.9)	<0.001
L1BMD	0.7 ± 0.2	1.0 ± 0.2	1.6 (1.2, 1.9)	<0.001
Spine BMD	0.83±0.16	1.06±0.19	0.02(0.19,0.67)	0.0466
Neck BMD	0.67±0.14	0.85±0.14	0.02(0.16,0.22)	0.02
Hip BMD	0.75±0.13	0.94±0.20	0.02(0.15,0.22)	0.000
HGI	0.5 ± 1.8	0.4 ± 2.0	0.0 (-0.2, 0.3)	0.699
DRINK			0.0 (-0.2, 0.3)	0.684
1	40 (36.0%)	102 (33.9%)		
2	71 (64.0%)	199 (66.1%)		
DPN			0.0 (-0.2, 0.2)	0.816
1	93 (83.8%)	255 (84.7%)		
2	18 (16.2%)	46 (15.3%)		
DKD			0.0 (-0.2, 0.2)	0.960
1	41 (36.9%)	112 (37.2%)		
2	70 (63.1%)	189 (62.8%)		

### 3.2 Correlation between HGI and bone mineral density and bone turnover markers

**Figure 1:**
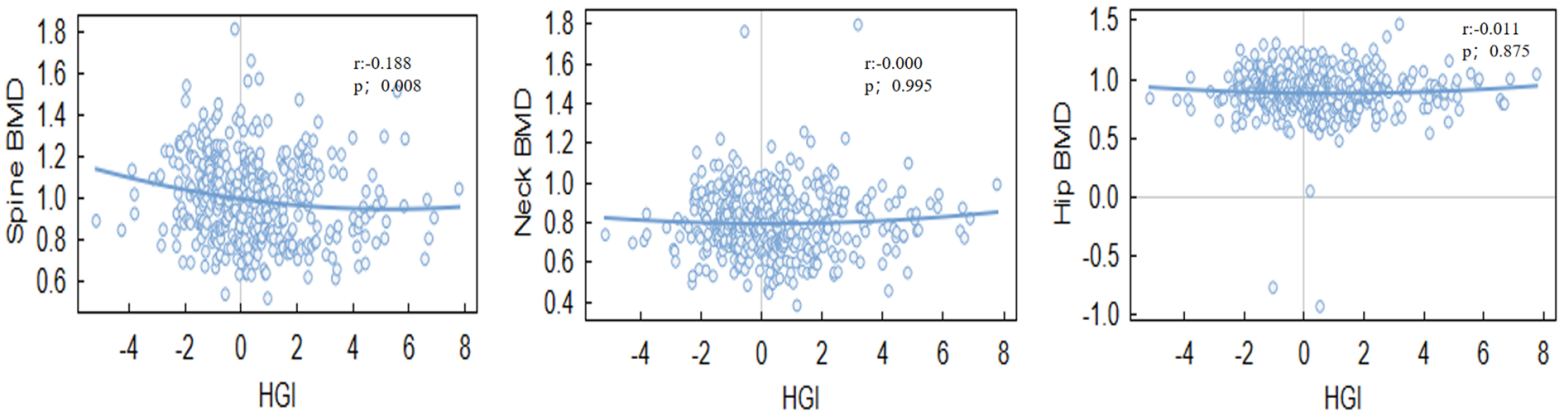
The correlation between HGI and bone mineral density at various sites.

**Figure 2:**
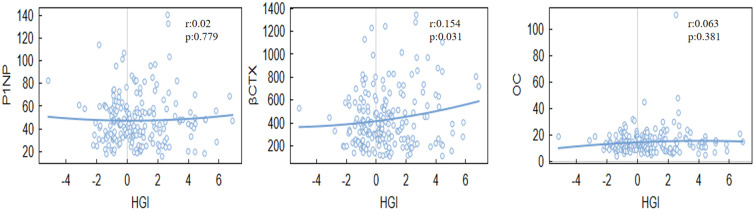
The correlation between HGI and bone turnover markers.

HGI was significantly associated with BMD at specific sites.Notably, a negative correlation was found between HGI and spine BMD (r = -0.140, P = 0.005), Even after adjusting for multiple confounding factors in the regression analysis, HGI remained significantly associated with spine BMD. while a positive correlation was observed between HGI and bone turnover markers, such as osteocalcin (OC) (r = 0.154, P = 0.030). However, the correlation between HGI and BMD at other sites, including the hip and neck, was not statistically significant (**Tables 2**, **3**). Among bone turnover markers, HGI showed a significant positive correlation with OC (r = 0.154, P = 0.030). However, no significant correlations were identified between HGI and PINP (procollagen type I N-terminal propeptide) or CTX (C-terminal telopeptide of type I collagen) (**Figures 1**, **2**).

**Table 2:** Demographic characteristics of the vertebral fracture group and the non-vertebral fracture group.

VF	1	2	Standardize diff.	P-value
N	72	340		
Age	66.6 ± 9.3	63.3 ± 8.6	0.4 (0.1, 0.6)	0.004
eGFR	109.0 ± 64.2	108.3 ± 38.5	0.0 (-0.2, 0.3)	0.910
Creatine	73.5 ± 27.2	71.9 ± 27.4	0.1 (-0.2, 0.3)	0.647
WC	90.7 ± 11.8	87.4 ± 8.6	0.3 (0.1, 0.6)	0.006
HbA1C	9.7 ± 2.4	9.1 ± 2.3	0.3 (0.0, 0.6)	0.020
FPG	9.2 ± 3.1	8.7 ± 3.1	0.2 (-0.1, 0.4)	0.148
TG	2.0 ± 1.1	2.0 ± 1.8	0.0 (-0.2, 0.3)	0.896
SHR	0.7 ± 0.2	0.8 ± 0.2	0.1 (-0.2, 0.3)	0.552
TyGindex	9.4 ± 0.7	9.2 ± 0.8	0.2 (-0.0, 0.5)	0.104
BMI	25.3 ± 3.6	24.5 ± 3.4	0.2 (-0.0, 0.5)	0.065
MOF	6.6 ± 3.4	3.4 ± 1.9	1.2 (0.9, 1.4)	<0.001
HF	3.0 ± 3.3	1.4 ± 2.4	0.6 (0.3, 0.8)	<0.001
PINP	48.7 ± 20.0	47.4 ± 22.0	0.1 (-0.3, 0.5)	0.772
Vitamin D	56.9 ± 21.3	53.6 ± 26.8	0.1 (-0.1, 0.4)	0.350
CTX	484.4 ± 320.3	425.2 ± 238.3	0.2 (-0.2, 0.6)	0.256
OC	14.8 ± 8.1	14.7 ± 9.8	0.0 (-0.4, 0.4)	0.984
Ca	2.2 ± 0.1	2.3 ± 0.2	0.1 (-0.2, 0.5)	0.386
AIP	0.2 ± 0.3	0.2 ± 0.3	0.1 (-0.2, 0.3)	0.582
L4BMD	1.0 ± 0.3	1.0 ± 0.3	0.0 (-0.3, 0.4)	0.827
L3BMD	1.0 ± 0.2	1.0 ± 0.2	0.1 (-0.3, 0.4)	0.621
L2BMD	0.9 ± 0.2	0.9 ± 0.2	0.1 (-0.3, 0.4)	0.711
L1BMD	0.9 ± 0.2	0.9 ± 0.2	0.1 (-0.3, 0.4)	0.624
Spine BMD	0.98 ± 0.24	1.00 ± 0.20	0.02 (-0.03,-0.08)	0.38
Neck BMD	0.76 ± 0.17	0.81 ± 0.16	0.05 (0.00,0.09)	0.03
Hip BMD	0.86 ± 0.17	0.89 ± 0.20	0.04 (-0.01,0.08)	0.16
HGI	0.8 ± 2.1	0.3 ± 2.0	0.2 (-0.0, 0.5)	0.096
DRINK			0.1 (-0.2, 0.3)	0.551
1	27 (37.5%)	115 (33.8%)		
2	45 (62.5%)	225 (66.2%)		
DPN			0.1 (-0.2, 0.3)	0.671
1	62 (86.1%)	286 (84.1%)		
2	10 (13.9%)	54 (15.9%)		
DKD			0.3 (0.0, 0.5)	0.027
1	35 (48.6%)	118 (34.7%)		
2	37 (51.4%)	222 (65.3%)		

**Table 3:** Correlation analysis between HGI and bone mineral density as well as bone metabolism markers.

		MOF	HF	Spine-BMD	Neck-BMD	Hip-BMD	PINP	CTX	OC
SHR	r	-.050	-.050	.145**	.014	.036	-.090	-.184**	-.158*
	p	.313	.310	.003	.784	.467	.208	.010	.026
TyG index	r	-.042	-.063	.132**	.128**	.119*	-.197**	-.116	-.244**
	p	.400	.205	.008	.009	.016	.005	.105	.001
AIP	r	-.013	-.027	.167**	.138**	.138**	-.191**	-.071	-.120
	p	.797	.592	.001	.006	.006	.009	.331	.103
HGI	r	.034	.031	-.140**	.024	.006	.022	.154*	.071
	p	.490	.530	.005	.630	.903	.755	.030	.323

*p < 0.05, **p < 0.001.

### 3.3 Mediation analysis

**Figure 3:**
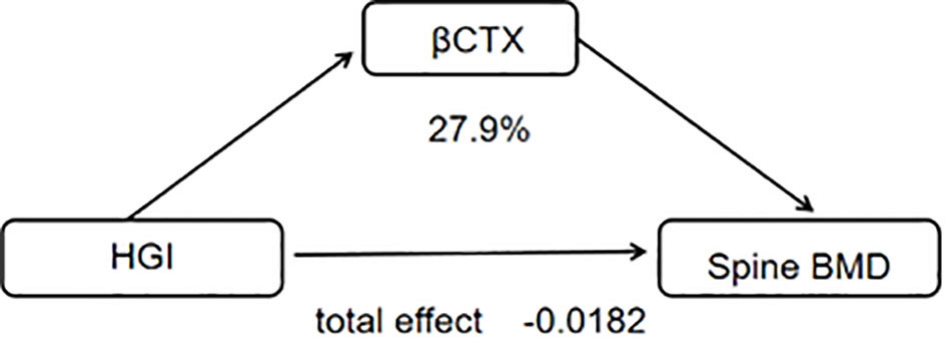
Mediating effect analysis of HGI on vertebral bone mineral density.

The mediation analysis revealed that HGI had a significant indirect effect on spine-BMD (ACME = -0.0052, P = 0.034). This finding indicates that HGI plays a mediating role in the relationship between specific variables and bone mineral density. Moreover, age-stratified analyses suggested that the influence of HGI on BMD varies across different age groups (**Table 4**; **Figure 3**).

**Table 4:** Mediating effect analysis of HGI on Spine bone mineral density.

	Estimate	P_value	CI	
			Low	Upper
ACME	-0.0052	0.034	-0.011	-0.0003
ADE	-0.013	0.054	-0.026	0
total_effect	-0.0182	0.008	-0.0316	-0.0044
prop_mediated	0.2792	0.038	0.0146	0.9013

### 3.4 Age-stratified analysis

Age-stratified analyses highlighted that the correlation between HGI and BMD was weaker in the younger group (<65 years) (r=–0.109, P=0.148)but stronger in the older group (≥65 years)(r= -0.185, P=0.005). This indicates that age may act as a significant moderator in the relationship between HGI and bone mineral density.

**Figure 4:**
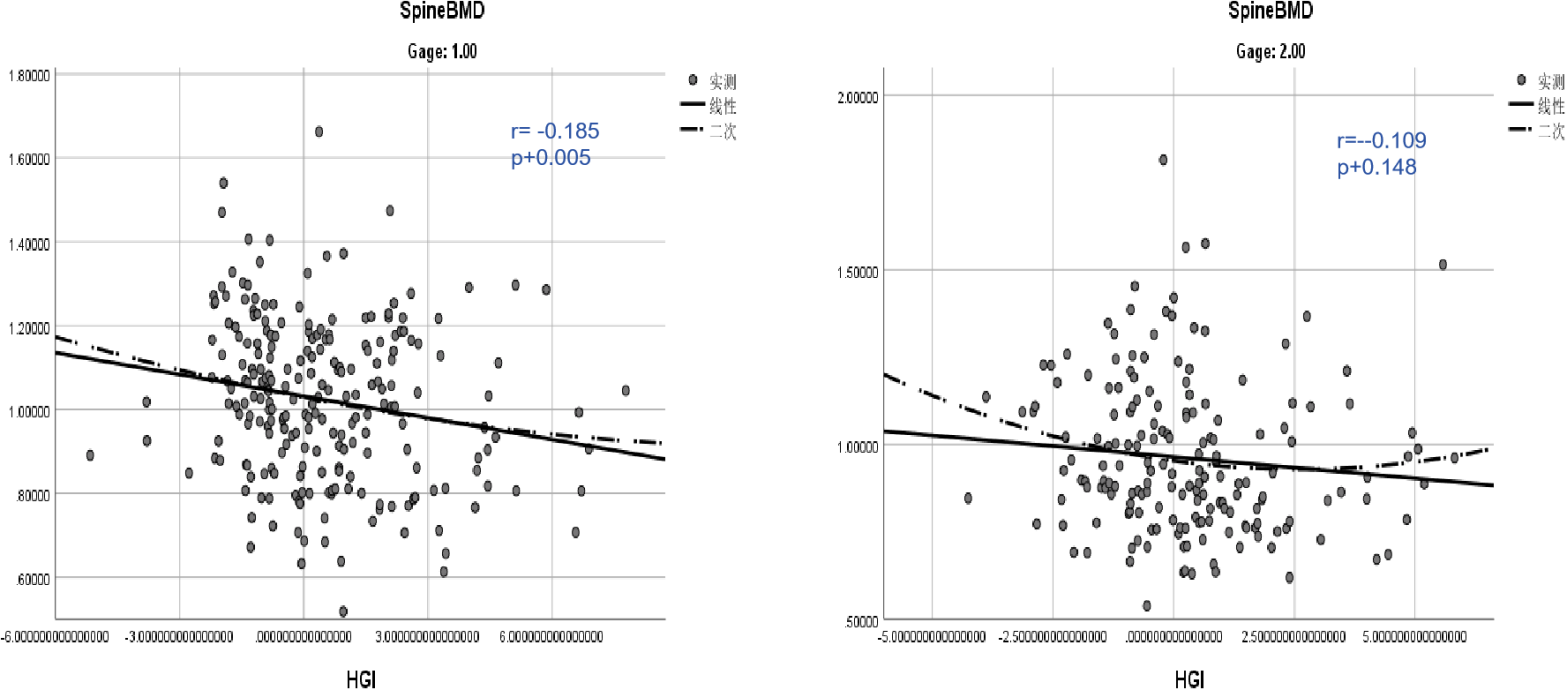
Age-stratified analysis of the correlation between HGI and vertebral bone mineral density.

In summary, this study demonstrates that HGI is associated with BMD and bone turnover markers, particularly spine-BMD and OC, and its effects are influenced by age. These findings provide novel insights into the potential role of HGI in bone health, warranting further investigation into its mechanisms and clinical implications (**Figure 4**).

## 4 Discussion

This study provides novel insights into the relationship between the HGI and bone health, particularly in the context of T2DM-related osteoporosis. Our results demonstrate that elevated HGI is associated with decreased BMD, particularly at the lumbar spine, and increased bone turnover activity. This underscores the importance of understanding the role of glycemic control in skeletal health, especially in populations vulnerable to osteoporosis, such as individuals with T2DM.

Specifically, the positive association between HGI and CTX indicates that glycation exacerbates bone resorption processes, The results align with previous studies on the relationship between glycemic control and bone health (21). For example, studies have shown that higher HbA1c levels are associated with reduced BMD and increased fracture risk (22). However, our findings diverge from earlier research that reported no significant correlation between glycemic control and BMD at certain skeletal sites. This difference may be attributed to differences in study design, population characteristics, or the inclusion of HGI as a more subtle measure of glycemic variability. By focusing on HGI rather than HbA1c alone, this study provides a more detailed understanding of how glycemic fluctuations specifically influence bone resorption and formation processes.

The observed associations between HGI and BMD can be attributed to several mechanisms. Chronic hyperglycemia, as reflected by elevated HGI, promotes the formation of advanced glycation end-products (AGEs), which accumulate in bone tissue and impair bone quality (23). AGEs disrupt with the collagen cross-linking process, leading to reduced bone strength and increased fragility (17). Additionally, AGEs contribute to oxidative stress and inflammatory responses, which further disrupt the balance between bone resorption and formation (24). However, our study extends this understanding by quantifying the mediating role of osteoclast activity in the relationship between HGI and BMD, a novel contribution to the field. Elevated HGI may also influence bone remodeling through hormonal pathways, including insulin and insulin-like growth factor (IGF-1), which are known regulators of osteoblast and osteoclast activity (25). Our findings, particularly the significant positive correlation between HGI and CTX, suggest that HGI primarily exacerbates bone resorption, contributing to reduced BMD.

However, the observed correlation between HGI and BMD appears to be site-specific, with stronger correlations at the lumbar spine compared to other skeletal sites, Several factors may contribute to this phenomenon: 1.Differential Bone Metabolism: The lumbar spine is metabolically active and is influenced by hormonal factors to a greater extent than other skeletal sites (26, 27). Studies have shown that glycation products can affect bone turnover and remodeling processes, which may have a more pronounced effect on BMD at the lumbar spine due to its higher metabolic activity. 2.Hormonal Regulation: Hormonal factors, such as insulin and advanced glycation end products (AGEs), play crucial roles in both glycemic control and bone metabolism (18). The lumbar spine is more responsive to hormonal changes, particularly those related to insulin sensitivity and AGE accumulation (28), which could explain the stronger association between HGI and BMD observed in this region. 3.Microenvironmental Factors: The microenvironment of the lumbar spine, including factors such as blood supply and mechanical loading, may influence the susceptibility of bone tissue to glycation-induced damage. The unique microenvironment of the lumbar spine could make it more sensitive to alterations in glycemic control (28), resulting in a stronger correlation between HGI and BMD at this site.

The site-specific nature of BMD also highlights the need for targeted approaches in managing osteoporosis, considering the specific characteristics of each skeletal site. Recent studies have made significant strides in osteoporosis treatment by developing targeted nanomaterials. For instance, bioinspired nanovesicles that target bone marrow endothelial cells (BMECs) have been reported to treat osteoporosis by converting the skeletal endothelium-associated secretory phenotype (29). These nanovesicles re-educate BMECs to secrete factors that promote osteogenesis and anti-inflammation, thereby improving bone quality and reducing fracture risk. Similarly, bone-targeted biomimetic nanogels have been developed to treat postmenopausal osteoporosis (PMOP) by scavenging RANKL and responsively releasing therapeutic PTH 1–34 in the bone microenvironment (30). These targeted biomaterials re-establish the osteoblast/osteoclast balance, enhancing bone quality. However, the specific effects of these treatments on different bone sites and their underlying mechanisms remain unclear. Our findings on the site-specific associations between HGI and BMD may offer valuable guidance for future research on targeted therapies and their applications in treating osteoporosis at various skeletal sites.

Recent advances have highlighted the role of biochemical markers of bone turnover in understanding the dynamics of bone metabolism under various glycemic conditions. Among these markers, CTX stands out due to its specificity in indicating bone resorption. Elevated levels of CTX have been associated with increased bone turnover, often leading to a decrease in bone mineral density (BMD)—a critical determinant of bone strength and a key diagnostic criterion for osteoporosis.

Our age-stratified analysis revealed a stronger association between HGI and BMD in older patients (<65 years), suggesting that age may amplify the impact of glycation on bone health (31). Aging is associated with reduced bone turnover capacity and increased susceptibility to oxidative stress and inflammation (32), which may amplify the effects of hyperglycemia on bone metabolism. This age-dependent relationship highlights the need for tailored approaches to osteoporosis prevention and treatment in older adults with diabetes.

This study has several strengths. First, it bridges a critical knowledge gap by investigating the role of HGI in bone health, a relatively unexplored area in osteoporosis research. Second, the use of multiple analytical approaches, including correlation, mediation, and age-stratified analyses, provides robust evidence for the relationship between HGI and bone health. The finding that osteoclast activity mediates a significant proportion of the relationship between HGI and BMD offers a potential therapeutic target for mitigating bone loss in T2DM patients. Lastly, our study highlights the importance of considering site-specific and age-related factors in understanding the complex interplay between glycemic control and bone metabolism.

Despite its strengths, this study has several limitations. First, the cross-sectional design prevents causal conclusions about the relationship between HGI and BMD. Longitudinal studies are needed to confirm the sequential order and causal pathways underlying these associations. Second, the study population consisted exclusively of hospitalized patients, which may limit the generalizability of the findings to broader populations. Third, while we accounted for key confounders, such as age, BMI, and comorbidities, residual confounding cannot be entirely excluded. Additionally, the lack of direct measures of AGEs and inflammatory markers limits our ability to fully elucidate the mechanisms linking HGI to bone health. Future research should incorporate these biomarkers to provide a more comprehensive understanding.

## 5 Conclusion

In conclusion, this study highlights the significant role of HGI in bone health, particularly in T2DM-related osteoporosis. By elucidating the mechanisms linking glycation to bone resorption and identifying osteoclast activity as a key mediator, this research provides a foundation for developing targeted interventions to mitigate bone loss. These findings underscore the need for a multidisciplinary approach to managing osteoporosis in diabetic populations, integrating glycemic control with strategies to enhance bone strength and quality.

## Data Availability

The raw data supporting the conclusions of this article will be made available by the authors, without undue reservation.
